# An expanded conceptual framework for solution-focused management of chemical pollution in European waters

**DOI:** 10.1186/s12302-017-0112-2

**Published:** 2017-03-09

**Authors:** John Munthe, Eva Brorström-Lundén, Magnus Rahmberg, Leo Posthuma, Rolf Altenburger, Werner Brack, Dirk Bunke, Guy Engelen, Bernd Manfred Gawlik, Jos van Gils, David López Herráez, Tomas Rydberg, Jaroslav Slobodnik, Annemarie van Wezel

**Affiliations:** 1 IVL Swedish Environmental Research Institute, PO Box 53021, 40014 Gothenburg, Sweden; 2 RIVM-National Institute for Public Health and the Environment, P.O. Box 1, 3720 BA Bilthoven, The Netherlands; 3 UFZ-Helmholtz Centre for Environmental Research GmbH, Permoserstraße 15, 04318 Leipzig, Germany; 4 Institute for Environmental Research (Biology V), RWTH Aachen University, Aachen, Germany; 5 OEKO-Institute for Applied Ecology, Postfach 17 71, 79017 Freiburg, Germany; 6 VITO-Flemish Institute for Technological Research, Boeretang 200, 2400 Mol, Belgium; 7 Unit H 01-Water Resources Unit, DG Joint Research Centre, Via Enrico Fermi 2749, 21027 Ispra, Italy; 8 Deltares, Postbus 177, 2600 MH Delft, The Netherlands; 9 EI-Environmental Institute, Kos, Slovak Republic; 10 KWR-Watercycle Research Institute, Nieuwegein, The Netherlands; 11 Department of Environmental Science, Radboud University Nijmegen, Nijmegen, The Netherlands; 12 Copernicus Institute, Utrecht University, Utrecht, The Netherlands

**Keywords:** Conceptual framework, Emerging pollutants, Solutions-focused, Prioritisation, Detection, Effects, Abatement, Mixtures

## Abstract

**Background:**

This paper describes a conceptual framework for solutions-focused management of chemical contaminants built on novel and systematic approaches for identifying, quantifying and reducing risks of these substances.

**Methods:**

The conceptual framework was developed in interaction with stakeholders representing relevant authorities and organisations responsible for managing environmental quality of water bodies. Stakeholder needs were compiled via a survey and dialogue. The content of the conceptual framework was thereafter developed with inputs from relevant scientific disciplines.

**Results:**

The conceptual framework consists of four access points: Chemicals, Environment, Abatement and Society, representing different aspects and approaches to engaging in the issue of chemical contamination of surface waters. It widens the scope for assessment and management of chemicals in comparison to a traditional (mostly) perchemical risk assessment approaches by including abatement- and societal approaches as optional solutions. The solution-focused approach implies an identification of abatement- and policy options upfront in the risk assessment process. The conceptual framework was designed for use in current and future chemical pollution assessments for the aquatic environment, including the specific challenges encountered in prioritising individual chemicals and mixtures, and is applicable for the development of approaches for safe chemical management in a broader sense. The four access points of the conceptual framework are interlinked by four key topics representing the main scientific challenges that need to be addressed, i.e.: identifying and prioritising hazardous chemicals at different scales; selecting relevant and efficient abatement options; providing regulatory support for chemicals management; predicting and prioritising future chemical risks. The conceptual framework aligns current challenges in the safe production and use of chemicals. The current state of knowledge and implementation of these challenges is described.

**Conclusions:**

The use of the conceptual framework, and addressing the challenges, is intended to support: (1) forwarding sustainable use of chemicals, (2) identification of pollutants of priority concern for cost-effective management, (3) the selection of optimal abatement options and (4) the development and use of optimised legal and policy instruments.

## Background

### The challenge

The increasing number of chemicals that are produced and applied in society represents a cause of concern for citizens, for the research community and for authorities. Lessons learned from legacy contaminants such as polychlorinated biphenyls (PCBs), mercury, and numerous others have created an awareness of the threats of widespread use of chemicals, yet without a comprehensive assessment of potential risks of all compounds and their mixtures. This awareness has led to the development of various regulatory instruments on national, European and global levels, but also an appreciation of the challenges that lie ahead of us to fulfil the ambition to develop a future sustainable use of chemicals.

There is widespread occurrence of man-made chemicals in the environment, and this occurrence is believed to contribute to losses of freshwater biodiversity and ecosystem services, e.g. [1–4]. Challenges remain to causally link the occurrence of specific polluting chemicals and chemical mixtures to the quality status of waters, to identify major chemical stressors, to identify emission and transport pathways, and finally to define solutions for the abatement of pollution-related risks and impacts. Complex mixtures of priority pollutants and emerging substances [5], transformation products [6] and natural compounds occur in aquatic systems, and the possibility to assess their combined effects is still limited [7–9]. Recently, the introduction of appropriate mixture assessment approaches was forwarded as a regulatory need, with a proposal to use a default model for assessing aggregated responses for a set of chemicals when more specific approaches still lack implementation [10]. The current capacity of analytical tools and models to fully and accurately handle the problem of net mixture exposures, hazards, risks and impacts of the mixtures of chemicals exposing man and ecosystems is insufficient and, therefore, needs improvement to judge whether a non-toxic environment has been reached, and if not, which water bodies and chemicals require priority attention for management. Also the development and efficient use of abatement strategies—technical and non-technical—for chemicals that are relevant today and in the future, and including the vast variety of mixture exposure situations, need to be advanced.

### Policy context and future needs for the advancement of the scientific basis for risk assessment and management of chemical pollution

In most cases, chemical-risk related policies are based on some form of risk assessment and prioritisation of individual chemical substances prior to their marketing and use, which then provides the basis for managing through labelling, restricting or banning the use of the most hazardous chemicals, preventing exposure or setting limit values for, e.g. contents in products or emissions to air and water. The procedures to identify which chemicals need to be restricted differ between legislative frameworks. Common principles include that they are based on utilising knowledge on their potential ecological or human health hazards, that the assessment is performed most often on single substances and that the final step is a combination of expected exposure and effects insights, with a political or administrative process where other priorities (e.g. socio economic or technical issues) may have an influence.

The main focus of the work presented here is on chemical pollution of aquatic ecosystems and topics relevant for the future development of the Water Framework Directive (WFD) of the European Union. The WFD is dedicated to the protection and restoration of a good ecological and chemical status of European surface waters. In the WFD daughter Directive 2013/39/EU, priority substances are identified and environmental quality standards (EQS) for them are defined. For priority hazardous substances, phasing out and cessation of emissions are required within a set time frame. Prioritisation is, therefore, a central operational concept in the management of polluting chemicals in the water environment in line with the WFD. Several different approaches for prioritisation are available, with different combinations of methods for risk assessment and ranking [11]. Risk assessment and prioritisation in the WFD currently are a site-specific- and retrospective approach and are, thus, based on concentrations of chemicals already present in the environment. For future development of the WFD, there is a need to develop more comprehensive methods to evaluate hazards and risks of chemicals and their mixtures as well as options to reduce and manage these risks.

Taking a broader perspective, the advancement of methods, procedures and tools for management of chemical risks is also potentially beneficial for the long-term vision of a non-toxic environment including non-toxic material cycles, as set out in, e.g. the Seventh Environmental Action Plan of the European Union (EU) [12]. On a global level, the UN program SAICM (Strategic Approach to International Chemicals Management) was initiated already in 2006 with the aim to achieve a sound management of chemicals throughout their life cycle to minimise adverse impacts on human health and the environment by 2020 [13]. The conceptual framework aims to provide the linkages between all options to forward safe chemical production and use, and eventually reaching the non-toxic status.

### The solutions-focused approach

The key characteristic of the solution-focused approach is to improve the utility of risk assessment outcomes by including an early evaluation of options for reducing risks [14–16]. This implies that the traditional steps included in the risk assessment (e.g. evaluation of risks for emissions, exposure, effects) need to be complemented with a structured approach to evaluate abatement options (technical and non-technical) as well as policy options for managing the problem. Following a solution-focused approach does not suggest that a traditional risk-based approach is abandoned but rather that available options for reduces risks should be evaluated in parallel with the overall purpose of providing solutions to potential risks at an earlier stage. As yet, there are few scientific publications demonstrating the use and usefulness of this approach in practical risk assessments, although the principle has been operationalised in the format of a solution-focused sustainability assessment [17], acknowledging that risk and sustainability assessments share similar process characteristics [18].

An increased interest in innovative, solution-focused approaches has also been under development in the USA to forward the development of a safe chemical economy [19, 20].

### The SOLUTIONS project

Research efforts to address the challenges outlined here are currently under way in the EU-funded project SOLUTIONS [21, 22]. The project focuses on a suite of approaches to evaluate current and future chemical emissions, (mixture) hazards, exposures, risks and impacts. A key motive for the project is to provide support to a shift from per-chemical risk assessment and management to a more holistic approach based on the solution-focused paradigm in risk assessment [15, 16] and the goal of a non-toxic environment.

### Aims

The challenges briefly described above have been translated in an effort to design a comprehensive conceptual framework for the assessment and management of chemicals.

In this context, the aims of this paper are:

1. To develop, present and discuss a conceptual framework for a comprehensive solutions-focused approach to manage chemicals and protect and restore aquatic environments.
2. To outline the main scientific challenges defined by the conceptual framework.
3. To summarise current progress and discuss recent and on-going research tackling these challenges.
4. To outline and discuss possible benefits and future use of the conceptual framework and the solution-focused approach to chemical management.

## Developing the conceptual framework

### Scope

The scope of the conceptual framework is broad and intended to meet the demands of future chemical management on several levels including identification of chemical risks via experimental tools and models, prioritisation and risk assessment as well as evaluating abatement options. The conceptual framework refers to WFD-specific needs and obligations and is intended for regional, national and international (EU) authorities and organisations responsible for implementing the WFD and developing river basin management plans. The conceptual framework also includes sections focussing on policy development, scenarios and future risks. These sections are included to take into account future needs for water- and chemical management and are, thus, also directed to a broader end-user group, i.e. involved in development of future chemicals policy and management criteria.

Apart from the WFD, the conceptual framework and the steps towards resolving the scientific challenges described here are also intended to provide support for other regulatory frameworks such as REACH [23], see discussion in “Providing regulatory support for chemicals management” section.

### Designing the framework

The conceptual framework was developed in interaction with stakeholders representing the drinking water sector, national and EU authorities, national and regional organisations responsible for managing environmental quality of water bodies and international conventions [24].

Stakeholder needs related to knowledge, tools and methods for assessment and protection of aquatic water ecosystems were collected by a survey and via further discussions at regular stakeholder board meetings organised as a part of the project. Topics included were, e.g. tools for monitoring and identifying drivers of chemical risks including mixtures, feasibility of applying models to estimate transport and ecosystem exposure to chemicals, technical and non-technical abatement options and requirements of a decision support system for access to knowledge on these topics.

The contents of the conceptual framework was thereafter developed with inputs from various scientific disciplines, covering emissions, chemistry, environmental chemistry, environmental contaminant modelling, ecotoxicology, human toxicology and abatement.

## The conceptual framework

### Overview and access points

A key characteristic of the conceptual framework is based on the recognition that the early attention on identifying options for management in the process of risk assessment improves the utility of the process. The solution-focused approach requires the development of guidance on how to apply assessment results to form a basis for decision making and development of policies and abatement strategies. Furthermore, guidance on how developed tools and models can be applied to address future challenges should be included. Finally, the solution-focused approach needs to be substantiated in terms of solution scenarios pertinent to the problem of concern, i.e. emissions and risks of chemicals in surface waters.

Here, an attempt to design a conceptual framework for assessment and abatement of current and future chemical pollution built upon the solutions-focused approach is presented, along with the necessary steps to operationalise the conceptual framework into practical use.

**Fig. 1:**
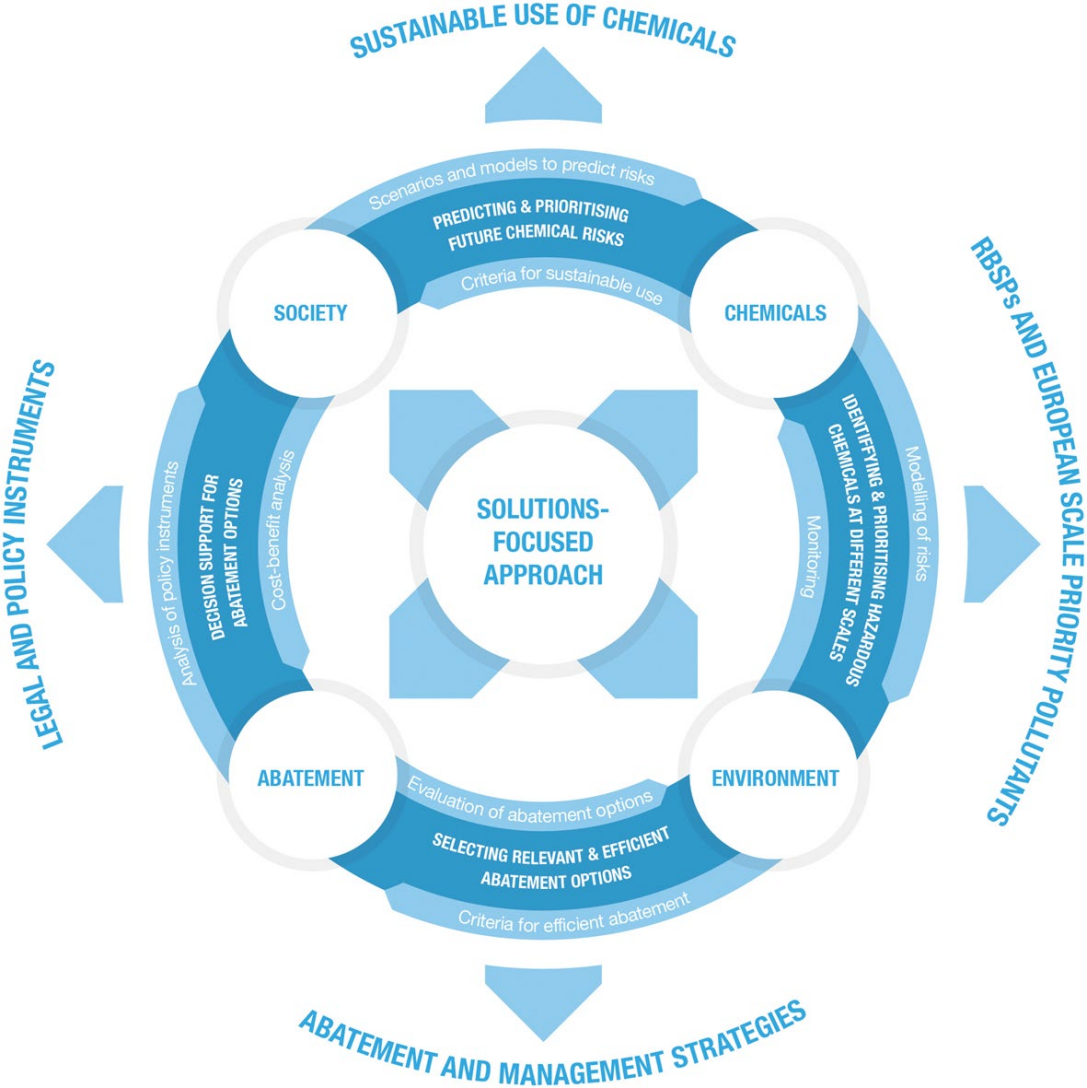
The conceptual framework for solution-focused assessment and management of the emissions, exposures, hazards, risks and impacts of chemicals and their mixtures related to human health and environmental impacts, with the different access points, derived from combining the solution-focused paradigm for risk assessments (central) with the specific problems of chemical threats. Modified from Brack et al. [21]

The conceptual framework presented in Fig. 1 has four main access points. The identification of the four access points was a direct consequence of evaluating the issue of contamination of aquatic ecosystems chemical in the frame of a solutions-focused paradigm: there is a suite of possible options to solve chemical problems, and these can be ‘organised’ according to the four major access points.

- The access point *Chemicals* defines issues concerning individual chemicals or mixtures that are produced, used or are predicted to be of relevance for the aquatic environment at the scale of a river basin or at an overall scale. In addition, it addresses criteria for a more sustainable production and use of chemicals in modern society.
- The access point *Environment* represents both aquatic environmental status and—with respect to drinking water and fish consumption—human health, and reflects contamination, ecotoxicity, toxicity and ecology again on the scale of individual river basins or at an overall scale.
- *Abatement* is the access point for compilation and evaluation of different abatement options, at any relevant scale. It relates to a set of abatement strategies that can be applied to a chemical exposure problem, ranging from technical solutions to reduce emissions (end of pipe filters) to substitution and non-technical approaches such as the spatial planning of emissions vis a vis vulnerable receptors (e.g. protected ecosystems or drinking water production inlets).
- The access point *Society* helps to account for and assess societal and political developments as well as policy instruments regulating production, use and emission of chemicals and the quality status of water resources. This also includes the availability and applicability of abatement options. One of the important aspects in the context of Society is also the field of risk communication.

The inclusion of four access points adds the entry points Society and Abatement to the two ‘classical’ ones commonly in use today: the Chemical as main entry point for assessments in chemical regulations (e.g. REACH), and the Environment (e.g. a water body) as main entry point of compartment-oriented regulations (WFD).

The development of the framework into a practical tool and guidance requires the fulfilment of the scientific challenges defined by the four interlinked subjects. This includes topics such as analytical tools for detecting and determining risks of chemicals in aquatic environments, modelling resources and results, abatement options, regulatory opportunities and scenario development. The framework presented here was also used as a template for the development of a user-oriented information support system (see “Guidance tools, databases and case studies”).

The basic conceptual outline depicted in Fig. 1 also provides the structure and framework for on-going research and development in the SOLUTIONS project for approaches that lack or are insufficiently validated [21]. The essential parts setting the agenda for the research relate to the linkages between the four entry points (circular arrows), and to the net types of contribution to a safe chemical use (outer circle: major output types).

The interlinkages between the four *access points* address the four *key topics* which describe the main challenges that can be addressed with the framework, both scientifically and in practice:

1. Identifying and prioritising hazardous chemicals at different scales.
2. Selecting relevant and efficient abatement options.
3. Providing regulatory support for chemicals management.
4. Predicting and prioritising future chemical risks.

The progress in fulfilling these challenges is elaborated below.

In a long perspective, the completion of these challenges will support goals related to improved environmental quality such as (1) *sustainable use of chemicals,* (2) *identification of priority pollutants,* (3) defining pertinent *abatement and management strategies* and (4) developing appropriate *legal and policy instruments*. The main focus of the work presented here is to describe the four overarching scientific challenges, the approaches to address them and the progress to date.

### Identifying and prioritising hazardous chemicals at different scales

The starting point of this topic is the Environment access point, and it concerns *evaluation of existing monitoring data: ecology, ecotoxicology, chemistry*. Depending on the quality and availability of existing data, gaps can be identified and used as a basis for development of a strategy for additional monitoring. The contamination of European water resources with a wide range of chemicals has been found to pose a significant risk to ecosystems [2–4]. Due to the large number of possible environmental contaminants, and the fact that they never occur as individual chemicals but always in more or less complex mixtures, the identification of potentially relevant chemicals and mixtures as well as their prioritisation for monitoring and abatement are key challenges for regulators and other stakeholders. This is an additional challenge beyond the assessment of impacted sites and individual chemicals emitted at a specific location (defined as River Basin Specific Pollutants) or of WFD Priority Pollutants defining the chemical status of water bodies. The conceptual framework presented here offers complementary topics to address this task: (1) *Integrated monitoring and whole mixture assessment* to detect chemicals and potential effects in different compartments including water, sediments and biota and (2) *Integrated modelling* to predict the transport, fate and risk to ecosystems and human health of defined chemicals based on information on production, use and emission patterns. The conceptual framework promotes a strong integration, interaction and validation of both approaches to achieve a most realistic setting of priorities.

In addition to assessing existing data, indications of candidate chemicals of relevance for future monitoring can be gained by *evaluating production, use, and emission patterns of chemicals* on the geographical scale of interest. Chemicals used in large amounts, or with specific use patterns that can be expected to cause emissions to the aquatic environment, or with high potency to cause harm, may be identified.

#### Improved tools for integrated monitoring and whole mixture assessment

Monitoring of the ecological status and chemical contamination is the backbone of the assessment of water quality according to the WFD with the intention to provide a holistic assessment on the way to a non-toxic environment. Currently applied approaches do not fully exploit the potential of integrated monitoring of chemicals and effects and thus do not fulfil the overall intention. Research in SOLUTIONS is aimed at advancing the existing monitoring toolbox with a specific focus on mixture assessment by developing and closely interlinking (1) advanced trait-based ecological tools and other in situ approaches using biomarkers in aquatic wildlife and caging experiments, (2) tools for enrichment of micropollutants from river water for subsequent biotesting and chemical analysis. (3) effect-based monitoring tools in vitro and in vivo, (4) multi-target-, suspect and non-target chemical screening as well as (5) advanced tools to identify drivers of adverse effects [22].

Linking the species occurrence and abundance data used for ecological status assessment with chemical contamination is yet a just a vision and challenge. Current approaches employ (i) multivariate analysis on existing monitoring data to allocate variance to different stress factors [25] and (ii) extend triad-based approaches of relating local habitat conditions with chemical contamination and in situ effects detected as biomarkers in aquatic wildlife [26] and in caged fish.

For integrated monitoring novel sampling tools are required to extract large volumes of water avoiding the logistic challenge of transporting them to the laboratory. Promising approaches include passive sampling [27] and active large volume solid phase extraction (LVSPE) [28].

Current chemical monitoring focuses on a small selection of chemicals and so does not consider all compounds that might occur, e.g. [29]. This invites improvements in monitoring of chemical concentrations to systematically improve coverage, e.g. [30] and the occurrence of potential mixtures. Thus, novel analytical screening tools using high-resolution mass spectrometry together with advanced software and workflows for data evaluation are developed [31–34] to address chemical contamination beyond well-known contaminants and priority pollutants and to characterise various water types for chemical profiles [35]. These non-target approaches are complemented by tailored analytical tools for compounds that might pose risks already at very low concentrations such as cytostatic drugs [36] and drugs of abuse [37, 38].

Effect-based tools have a great potential to monitor chemical contamination on the basis of its interaction with standardised biological laboratory systems indicating the exposure to chemicals with a specific mode of action (MoA) or effects on survival, growth or reproduction of aquatic organisms. In contrast to current chemical monitoring, effect-based tools provide measures for the load of an environmental compartment with all chemicals exhibiting an effect on a toxicological endpoint and thus reduce the risk that major contributors to risk are overlooked. However, a careful analysis and prioritisation of MoAs is required and has been made by Busch et al. [5] for about 1000 chemicals that are frequently detected in European water bodies. More than 50 effect-based tools addressing prominent MoAs are under evaluation with individual chemicals, defined mixtures and environmental mixtures. In several case studies, panels of these tools were tested successfully for their applicability on environmental samples [39–41]. A key component for the identification of hazard drivers in complex mixture exposure is the EDA approach, where combinations of non-target/target screening [31] with bioanalytical tools can be applied in different tiers to identify drivers of toxicity or in combination with toxicity information or hazardous properties of encountered substances [35, 42]. Stepwise approaches for the use of bioanalytical tools, which details the developing methodologies for innovative monitoring also including mixtures, have been presented [5, 22, 43].

Responses in effect-based tools may result in the requirement to identify the drivers of these responses to address these chemicals with further assessment and management. Mass balance approaches [40, 41] and effect-directed analysis (EDA) [44] have been identified as key tools for driver identification combining biotesting and chemical analysis. Complexity is reduced with fractionation and/or multivariate statistics to link chemical signals to adverse effects. An in-depth overview on EDA tools has been provided in [43].

The confirmation of substances identified after non-target screening, including information on intrinsic hazards remains a challenging task. It is complex to link observed toxic effects in complex environmental mixtures to responsible toxicants in EDA, and subsequently to abatement measures. Non-target screening using novel instrumental techniques in combination with compound databases and *multi criteria analysis* has been shown to be a powerful approach for identifying candidate substances contributing most to the chemical risk in a water body [45].

#### Observable effects, candidate chemicals and sites of interest

Applying improved monitoring tools provides the means to identify a first selection of observable effects, candidate chemicals and sites of interest when applied on, e.g. basin scale. Current monitoring and the innovative approaches taken together can provide a clear spatio-temporal ranking across samples in terms of frequency and degree of exceedance of criteria (if available for measured chemicals), combined with exceedance of (independent) effect-related signals. When such data are further collated into (bio)monitoring data sets that expand large regions, similar rankings can also be obtained via diagnostic, eco-epidemiological methods which look into the occurrence or abundance of species in relation to multiple stress [4]. After the initial ranking, based on monitoring, modelling or eco-epidemiology, or both, the aforementioned tools can be used for *in*-*depth confirmation of the priority ranking of compounds and effects* via site assessment using effect-based diagnosis, non-target effect studies, higher tier EDA and biomarkers.

#### Integrated modelling

**Fig. 2:**
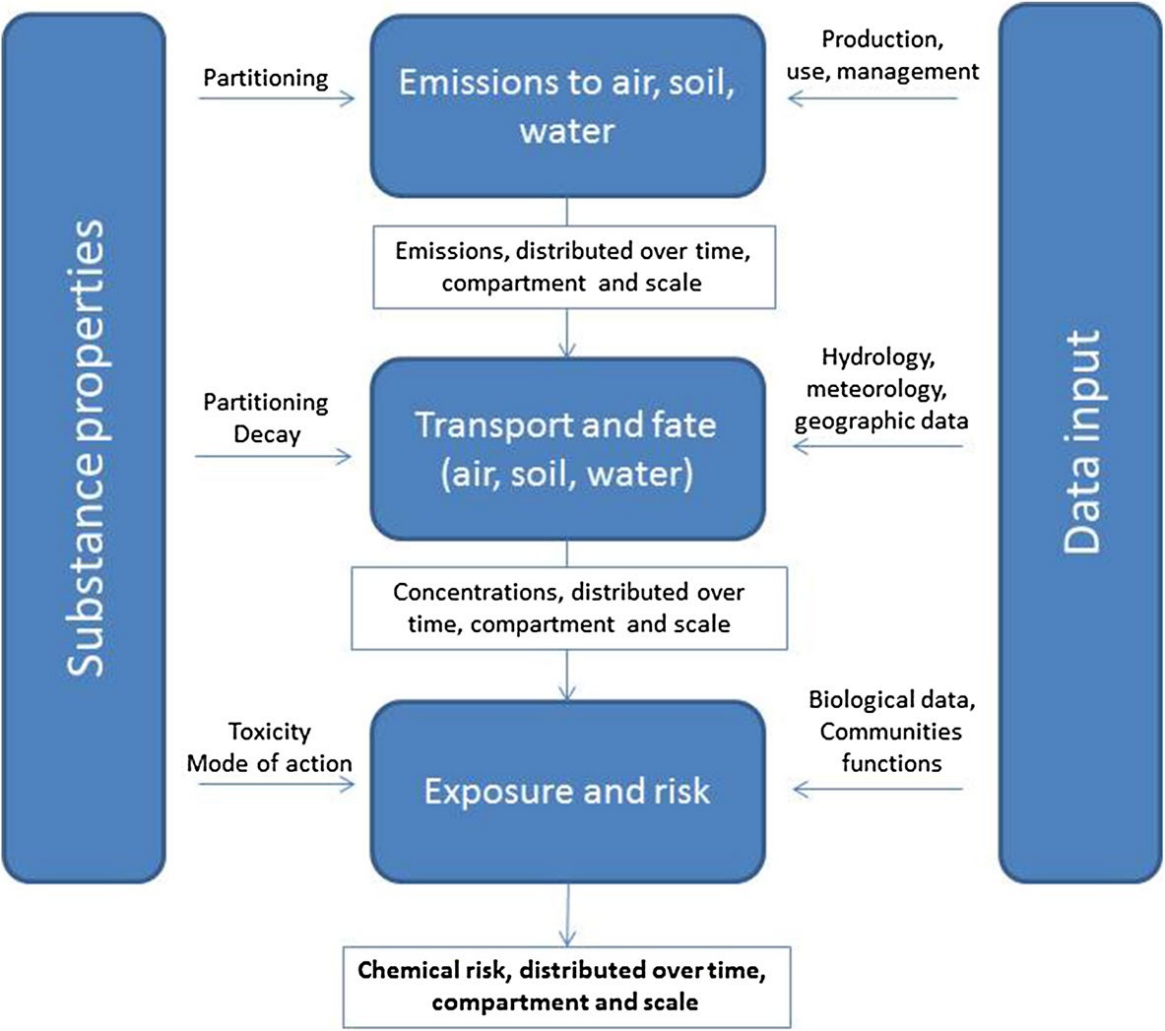
Structure and function of the steps of an integrated ‘model train’ that can be used in a versatile manner to address management problems in water quality management, and that combines models and data on chemicals (regarding emissions, fate and behaviour) with those on sensitivity of exposed organisms and other relevant data (e.g. on the hydrology of European surface waters)

Starting from the entry point ‘Chemicals’, but closely associated with the entry point ‘Environment’, SOLUTIONS focuses on creating and using an integrated ‘model train’ (Fig. 2) to quantify expected mixture impacts, and trace those back via risk assessment, exposure assessment and eventually emitted masses of chemicals. Modelling is the only way to explore the meaning of the potential emissions of chemicals that will be produced in the future [46], and thus help deriving abatement strategies ‘before the event’.

Models provide a useful tool to fill data gaps and to predict fate and effects for compounds which cannot be identified or have not yet been identified using the analytical tools described above. Models are a basic tool for extrapolating over larger geographical areas, to provide generalised results on varying chemical exposure patterns in space and time [47–51]. Model results can be designed to inform authorities on priority of sites, priority of contributing substances and on probable effect types. Examples of large-scale predictions of environmental concentrations of many chemicals have already been presented in [52, 53].

Apart from the widely used standard emission scenarios utilised in the safety evaluation of chemicals, the use of more spatio-temporally explicit modelling results in decision making is still a promise rather than a standing practice. Modelling results can also complement interpretations based on environmental monitoring data and provide information on the consequences of predicted emissions and occurrence in water systems. Together, monitoring and modelling can provide a basis for deriving hypotheses on potential priority causes of impacts and candidate chemicals for risk reduction planning, as well as for further dimensioning monitoring and modelling activities.

The integrated modelling approach still encompasses a number of challenges, which are currently the focus of the research and development. These include generation of emissions data, linking of models for different physical compartments as well as to development and application of models for effects and risks. Significant steps towards a full model train—to explore potential impacts starting from production volumes and chemical identities—have been made already. For compounds regulated under REACH, the collation of production data, and the derivation of the fraction of those compound emitted to the environment via environmental release category (ERC) data, has yielded insight in compound-specific quantities that potentially reach the environment, especially water, and in a basis to derive predicted environmental concentrations [54], vastly expanding on an earlier study focusing on the so-called high-productive volume chemicals only [52]. Especially, persistent and mobile substances appear to be problematic [55].

Chemical fate studies building forth on emission data or models highlight the spatial variability of chemical concentrations, as exemplified for case studies focusing on perfluorinated compounds and the Danube catchment [56]. Such analyses serve as a basis for an extension towards European-wide analyses, accounting for hydrology-based fate and transport modelling [50, 51]. Regarding the characterisation of ecological risks and expected impact magnitudes and site rankings, substantial progress has been made in terms of the number of compounds for which concentration-impact information has been collated (>2000 compounds), expanding more than tenfold over currently known data of this kind [57].

Models can in principle be applied for a large set of chemicals and provide support to prioritisation at varying scales, within or across compound groups, and for various abatement alternatives. Evidently, each step in modelling the derivation of final risk- or impact rankings needs to be evaluated and validated, e.g. by comparing predicted and monitored environmental concentrations, and predicted impact magnitudes with observed biodiversity changes. This approach was used to explore the expected mixture impact levels given by the application of >250 plant protection products in the Netherlands, and yielded insights in predicted priority sites as well as priority compounds and also included validation of these outcomes by comparisons to observed presences and abundances of species [58].

Results from the measurement and modelling approaches described here are expected to enable establishment of *proposed European and basin scale key toxicants,* which are defined based on scientific evidence. Apart from local or catchment-specific measures taken in the context of river basin management, the forwarding of proposed priority chemicals to a legal framework, i.e. to the list of priority substances under the WFD, a political process involving negotiations between member states is needed.

### Selecting relevant and efficient abatement options

The solution-focused approach has added the novel entry point ‘Abatement’ to the conceptual framework, in a position of potential application early in the assessment process. By this early integration of risk assessment with risk management a ‘learning system’ that includes a continuous evaluation of abatement options, scenarios and effects can be achieved. Following from the results from identifying and prioritising hazardous substances, the approach here is to identify, document and store a wide range of possible (existing) technical and non-technical abatement options [17, 21], and to develop and use an intervention database as well as guidance and decision support on how to select between abatement options. The role of both local and regional- or catchment scale stakeholders in the early generation of potential abatement scenarios is a key characteristic of the assessment and management process here. This also includes the involvement of stakeholders that are emitting chemicals and make use of the water system, requiring high quality water, such as agriculture including greenhouses, industry, the care sector and households.

The first step for prioritising abatement options is based on the results from the approach described in the previous section, i.e. defined and prioritised chemicals and water bodies for abatement. In addition to this, an *assessment of emission sources and patterns* is necessary for the identification of both the type and possible location of priority emission sources causing the priority sites, to focus abatement. This process can be further supported by a systems-level approach tracking of groups of substances from production, use, emissions, fate and end-point, to provide or confirm the relevant information for selection of abatement alternatives and for assessment of placement strategies.

#### Prioritised and ranked abatement options

A compilation of existing abatement in the form of *Intervention Database* is under development [59]. This database will include information on both technical and non-technical abatement options based on qualitative and quantitative experiences of implementing abatement techniques gained so far. The approach in handling this database allows for continuous expansion of the abatement options, as well as their efficacy, as experiences with abatement approaches and efficacies grow. The non-technical abatement options may include ongoing advancements in, e.g. the development of green chemistry, substitution, ban and use restrictions of priority chemicals, as well as issues like education of producers and consumers. For technical abatement options, the database contains removal efficiencies of chemicals for given abatement techniques, which may thereby be expressed for indicator chemicals, but also for chemical mixtures and with links to effects as based on bio-analytical tools. Estimated or actual costs of each measure will be given room in the database. As the database contains abatement options which are taken by various sectors (such as health care, agriculture, industry, consumers), the concepts embedded in the database will also facilitate cross-sectoral learning and identification of novel abatement options. In addition to the contents described above, the database will also contain information on stakeholders and their potential roles in both causing and abating emissions. This will support the development of management options and to assess the ease of implementation of the selected abatement option and also to allow identification of “low hanging fruit”.

In combination with the modelling train, simulations of expected effects of both the type and location of different abatement scenarios can be made, and the results can be used to optimise measures on a basin scale. An example of this approach is the study of pharmaceutical removal from wastewater treatment plants in the Netherlands [59]. In this study, the spatial association of wastewater treatment plants in relation to sensitive functions of the water bodies, e.g. drinking water production and nature reserve areas, was used to discuss a spatially relevant and cost-effective abatement strategy.

In the future, the quantitative data on removal efficiencies in an expanding database can offer further opportunities for optimising abatement. That is, with sufficient data coverage, the removal efficiencies in technical abatement options can be evaluated against *chemical properties* (physico-chemical) of selected chemicals, to allow the derivation of generalised results useful for emerging substances not yet identified. Innovative possibilities to develop a *‘QSAR for (technical) abatement efficacies’* may thus be explored [60].

### Providing regulatory support for chemicals management

The access point ‘Society’ allows the introduction of the existing set of regulations, each covering part of the realm of chemical emissions, exposures, risks and (current) abatement obligations into the risk management process. The access point also points at subjects like risk communication and the political system and context in which decisions are taken. By considering the societal flow of chemicals (Substance Flow Analyses), the overall aim of the analysis of this topic here is primarily to evaluate current regulatory contexts, so as to provide guidance on existing and possible future policy frameworks to stakeholders.

#### Overview of relevant policies for hazardous emerging chemicals

Chemical pollution and reducing risks to the environment and human health are the focus of a large number of EU directives regarding, e.g. drinking water, specific products and handling of waste. Chemicals are also managed within a number of regional and global conventions such as the Stockholm Convention on Persistent Organic Pollutants. Most, if not all, directives and conventions include routines and requirements for reporting and collecting information, monitoring and implementation, but with differences in focus and ambition level.

Synergies between the WFD and REACH—the main chemicals regulation in the EU provides potential for future improved efficiency of regulatory actions. By combining knowledge on risk assessment and prioritisation from the WFD, which are based on monitoring and evaluation of presence and impacts of chemical contaminants already present in water ecosystems, with the prospective approach of regulating use and emissions of chemicals in REACH, more efficient risk management can be achieved.

A synopsis providing short descriptions of the different regulations, directives and multilateral environmental agreements and information about the substances they regulate is already available in a database [61]. Geiser [19] provides a worldwide overview of regulations specifically aimed at chemicals.

For the European situation, the compiled information serves as input to an evaluation of potentials for synergies between the WFD and other policies and conventions focusing on chemicals. The evaluation focuses on potentials for exchange of information on, e.g. use, emissions, monitoring results, abatement options, prioritisation and/or risk assessment methodologies but also on synergies in terms of implementing measures. The latter includes an analysis of the procedure for including new chemicals in the policy framework as well as the efficiency of action taken under the framework, i.e. how efficient is the policy to go from identification of a problem to solution.

The analysis also provides an identification of gaps and inconsistencies in current policies which can be in the form of groups of chemicals or specific sectors in society not or poorly covered by existing legislation.

**Fig. 3:**
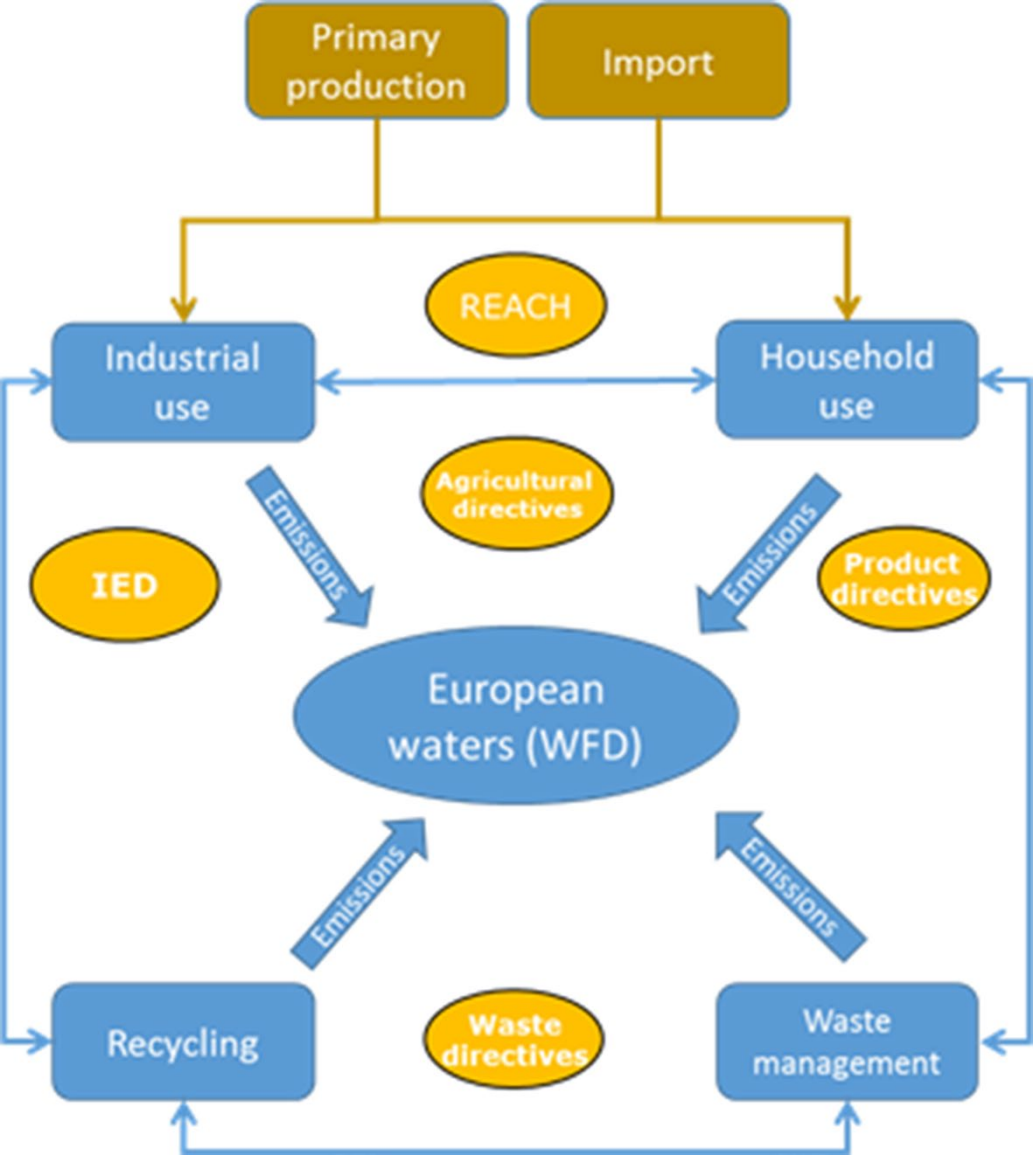
Schematic substance flow analysis for chemicals including examples of where policies interact in the system. From different use (*blue coloured objects*) of produced or imported chemicals (*brown coloured objects*) and the emissions generated to the receiving waters and were in this system different policies interacts (*yellow objects*). *IED* Industrial emissions directive, Directive 2010/75/EU on industrial emissions; *REACH* Regulation concerning the Registration, Evaluation, Authorisation and Restriction of Chemicals (REACH), Regulation (EC) No 1907/2006

The analysis of the regulatory system is performed in relation to the production, use and releases of the chemicals in society, i.e. following a substance flow analysis (SFA) approach [62, 63]. The purpose of developing an SFA is to describe the main flows of chemicals in society from production (or import), via industrial use, consumption in households, recycling and waste management.This information is necessary for identification of where in the societal life-time of the chemical, interventions in the form of policy restrictions or abatement can be applied to avoid or reduce emissions. Also for evaluation of where the intervention affects a large mass flow for different alternatives for up-stream measures, substitution or end-of-pipe removal. A schematic graph of an SFA is presented in Fig. 3, where also the possible intervention points for polices and abatement measures are represented. For a specific chemical, each of the flow paths described in the figure can consist of a number of different compartments and sub-paths, as well as interchange between different compartments in, e.g. industry. Furthermore, emissions will occur not only to European waters, but also to air, soil, etc.

The results of the analysis of policies will, in combination with the assessment of abatement options, form the basis for providing advice on applicability of *National and European legal and policy instruments* to phase-out or restrict chemicals or groups of chemicals. The results will also be evaluated in relation to gaps to provide insight into the prospect on developing a future *innovative regulatory framework* for substances of emerging concern. These recommendations will be made in relation to new scientific knowledge such as effects of mixtures and results of applying novel analytical tools for occurrence and effects as well as new developments in society affecting releases, e.g. population growth and demographic distribution and associated new consumption patterns for pharmaceuticals and biocides as well as an increased material recycling in the envisaged circular economy.

### Predicting and prioritising future chemical risks

The identification of future risks, and more specifically the chemicals of tomorrow that will or may cause future risks, is an evaluation that is needed but also associated with large uncertainties as it is driven by a multitude of pressures. More than 100,000 chemicals are used in society and predicting future trends and changes in their use is complex. Furthermore, changes in policies and regulations will affect future use and releases of chemicals to an unknown extent. The problem of chemicals can not only be addressed via typical response types (as used in the specific bioassay approaches sketched above), but also via emission profiles. A profitable strategy can be to explore whether emissions of harmful chemicals to a water body can be quantified via typical emissions profiles for various sectors and/or geographical area based on geographical data, population, consumption patterns and specific descriptors for industrial and agricultural activities. Such a profile would consist of a suite of chemicals of a typical composition and a range of properties. Combining this approach with modelling may elucidate at which level the aggregated emission profiles become hazardous.

#### Identification of current use of chemicals in industry/society

The starting point of this topic is results from evaluating existing monitoring data (ecology, ecotoxicology, chemistry) and production, use, and emission patterns of chemicals on the geographical scale of interest as well as a survey of the existing policy framework [62]. This information can be compiled to provide an integrated baseline of the current status relating to use sectors, emissions, regulatory options and current environmental status of chemicals.

#### Future risks based on economic, technical and policy development

The approach taken to address future emissions is, starting from the baseline described above, to *assess relevant scenarios (e.g. concerning economy, demography, industry, consumption, energy, agriculture, hydrology)* and analyse how these scenarios may affect use and emissions of chemicals. To date, more than 30 scenarios for developments in society have been identified addressing medium- and long-term developments in society, predictions for water use and water cycle, developments due to climate change, to demographic change and others. Many scenarios addressing potential developments in society have been developed and presented but only in a few cases implications of the predicted developments on emerging pollutants are mentioned explicitly. More frequently, general predictions can be found, e.g. regarding future water consumption, food production and consumption behaviour. In some cases, it is possible to use these general predictions to draw conclusions on potential future developments of contaminants (e.g. increase in crop production and associated increase in the amount of pesticides used). Four sector-specific areas have been identified in SOLUTIONS as important for future chemical pollution: health care, food production, new materials and urban growth. Megatrends such as climate change and demographic change are included as drivers in these sector scenarios.

New pathogens, longevity, increase in animal farming, growing cities and sprawl, urban mining and increased use of new technologies are examples of developments which may influence chemical use and emissions and are occurring already now and will continue in the next decades. Patterns of use will change in future for pharmaceuticals, biocides, household chemicals as well as for specific groups of industrial chemicals. In many areas, legacy chemicals will contribute significantly to the future impact on the environment—besides unexpected new substances.

Some of the future trends can be integrated in exposure and risk modelling. Examples are predictions on demographic change and changes in the consumption pattern of pharmaceuticals. Other trends can have implications for effect monitoring. The expected increase of emissions of pharmaceuticals could stimulate the monitoring of drug-specific endpoints, e.g. behavioural changes in fish. One of the key natural trends, partly related to water use by man, is the hydrology of a system. The hydrology determines to which extent emitted chemicals can be diluted, to which extent water is used and re-used by man for (sensitive) purposes and, thus, to which extent high concentrations occur given low river discharges and water scarcity. Various global and regional models stress the massive importance of considering hydrology and water use as major co-driver of net exposure concentrations to chemicals [64].

### Guidance tools, databases and case studies

Experience has learned that mixture exposure situations are highly diverse throughout Europe: contamination levels range from negligible to low, concentrations from low to high, mixture composition from simple to complex, and exposed ecosystems from small stagnant waters to large rivers. Moreover, the management problem and the abatement options vary, on a site-specific basis. Acknowledging this variability resulted in the idea to think in the concept of a versatile toolbox rather than in a few fixed approaches. The following major components of the toolbox are seen:

- A meta-model (RiBaTox) which will enable identification of approaches and tools to address a wide variety of water quality management problems;
- An integrated data portal (IDPS), which enables storage, retrieval and use of data required for using the tools collated in RiBaTox.

#### RiBaTox—meta-model for assessments

The major objective of the RiBaTox development is to provide a user-friendly computer tool to find the pathways to solutions to stakeholder problems on assessment, prioritisation and abatement of environmental mixtures of (emerging) toxicants in water resources. This tool, called RiBaTox, is based on the practical implementation of the Conceptual Framework and will guide the end-user to the appropriate models, tools and databases to help solving the stakeholders’ problem.

RiBaTox is being developed as a freely available web service. It will interact with the end-users through an internet web browser accessible via the public web-page of SOLUTIONS and the Integrated Data Portal for SOLUTIONS (IDPS), usable by the intended end-users at the national and local levels.

#### Integrated data portal for assessments

Within the SOLUTIONS project, the development of the IDPS will act as an interlinked portal for the knowledge-base of the (current) research consortium and future users. The idea is to build up a centralised infrastructure for discovering and accessing information or information resources on priority and emerging pollutants in land and water resources management. The information includes compound and structure-specific property and toxicity information, geo-referenced monitoring data, receptor-specific data on traits that may be affected and spatial data on human population, land-use, geology, hydrology and climate that impact the emission, transport and fate of pollutants. Moreover, the platform aims to support a coordinated approach for collection, storing, accessing and assessing data related to present and future emerging pollutants.

#### Validation and case studies

To ensure that the research and development in SOLUTIONS are useful and applicable to solving problems related to emerging pollutants in European waters, three case studies are included in the project: the Danube, the Rhine and the Iberian rivers Ebro and Llobregat. These case studies cover specific aspects of water contamination and management problems, whereby the Danube is an example representing a large transnational catchment, the Rhine a catchment in which ongoing abatement strategies can be evaluated, and the Ebro and Llobregat catchments with substantial hydrological dynamics and water shortage.

These approaches and strategies are currently being applied and explored using case studies in the Danube and Rhine river basins as well as for rivers of the Iberian Peninsula. Currently, the first results from analytical and bioassay studies are being compiled and evaluated [40]. A synthesis of findings will be organised to provide guidance for future solution-focused environmental monitoring and explore more systematic ways to assess mixture exposures and combination effects in future water quality monitoring.

In the Danube, the studies are performed in cooperation with the International Commission for Protection of the Danube (ICPDR) within the framework of the third Joint Danube Survey (JDS3) conducted in 2013. This cooperation has provided a unique opportunity to validate all components of the integrated risk modelling, to explore cause–effect relationships, and to suggest RBSPs with respect to ecosystem and human health. This first draft list of Danube River Basin Specific Pollutants was included into the 2015 update of the Danube River Basin Management Plan [65] and it helped to fill in a significant gap in addressing pollution by hazardous substances which is a significant water management issue in the international Danube River Basin District. SOLUTIONS’ direct contribution to the WFD implementation in an international river basin is also a good example of practical application of science-policy interface allowing researchers to communicate scientific findings to policy-makers. JDS data will also be exploited to support higher tier ecological risk modelling, addressing a multi-stressor situation. The project targets include suggesting a list of the Danube RBSPs together with their draft EQSs, which will be discussed with all stakeholders. A recent study on the Danube has shown that the species inhabiting the river are subject to multiple-stress conditions, whereby chemicals are a significant driver of impacts, together with other stressors [25].

In the Rhine case study, the focus is on abatement strategies. A number of European utilities in Switzerland, Germany and the Netherlands are considering implementation of additional technologies to enhance the removal of emerging compounds in wastewater effluents such as advanced oxidation or activated carbon treatment. A comprehensive trans-national cost-efficient placement strategy of abatement options to improve surface water quality will be developed in the Rhine case study utilising both new information on occurrence and impacts of emerging substances and comprehensive hydrology-based modelling approaches combined with geospatially specific emission patterns.

In Ebro and Llobregat, water scarcity, enhanced by climate change, provides new challenges for maintaining a good water quality, as e.g. intensive use of pesticides in agriculture have currently been described in [66]. In this case study, models and tools will be applied on the river basins Ebro and Llobregat to provide significant synergies with earlier findings which offer a unique case complementary to the Danube and Rhine studies.

The case studies—although currently in operation—have already been providing a valuable input to the practical testing of the conceptual framework, so that a consistent and practice-oriented guidance for the early detection, identification, prioritisation, and abatement of chemicals in the water cycle can be produced. A regular feedback by stakeholders such as ICPDR [67] and ICPR [68] ensures that a pragmatic approach is being developed for solving stakeholders’ problems with emerging pollutants in line with the EU legislation.

## Discussion

### Improved solution focus

The development of a practical application of the conceptual model will deliver a number of products that can be used for the implementation of approaches forwarding a solutions-focussed approach for chemical risk assessment and management in Europe.

Building on the project’s tools, guidance documents and experiences compiled in the decision support system (RiBaTox) and the extensive databases with data on occurrence, effects and properties of chemicals (IDPS) it will be possible to move towards a true solutions-focused risk assessment as defined [14, 16].

### Evaluating and communicating solution-focused scenarios

**Fig. 4:**
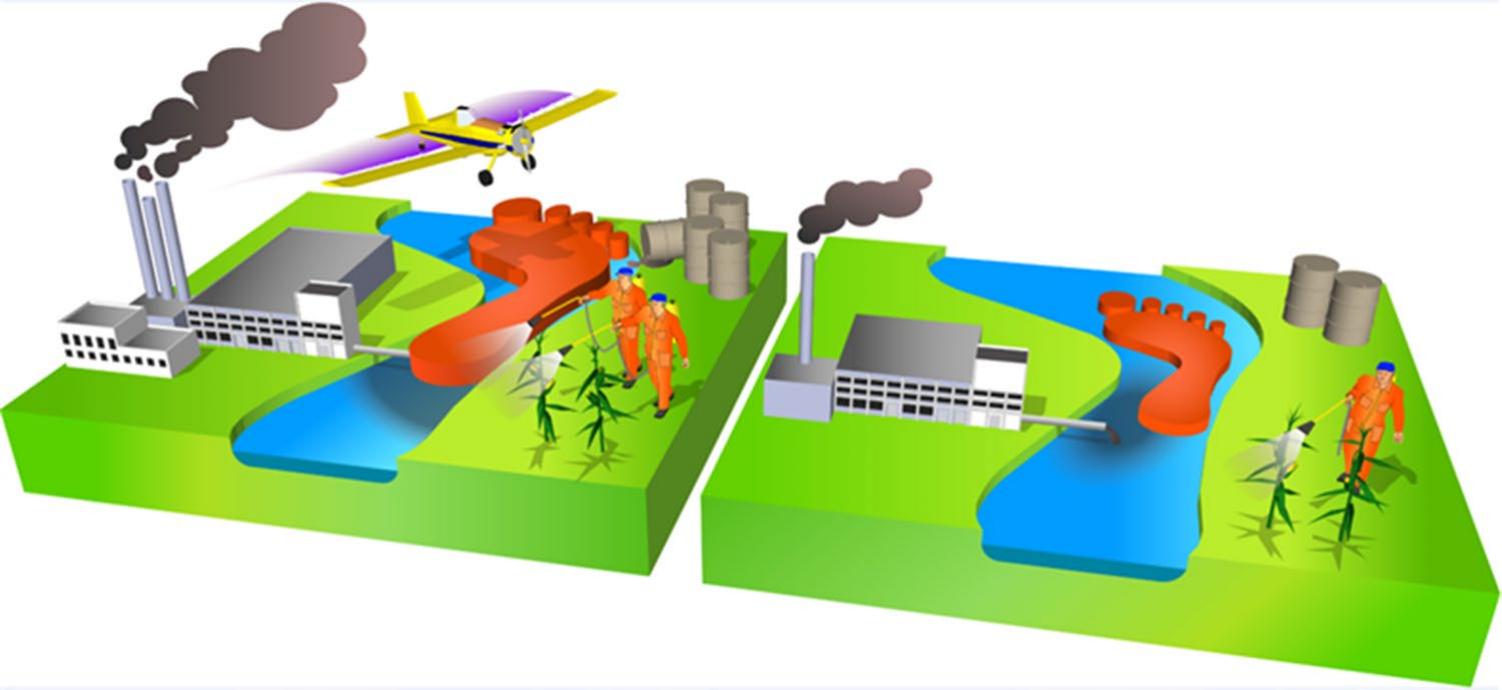
Scheme of communicating outcomes of mixture risk assessments for a specified region (area, with its water bodies) by chemical footprinting (i.e. checking whether the volume water needed to dilute chemical emissions to a safe level is available in the region), represented as the ‘foot’ size versus the water volume, and the effect of (multiple) abatement strategies (from *left to right*: footprint reduction as a result of various abatement strategies). Reproduced from Posthuma et al. [57]

Chemical pollution has been proposed as one out of nine so-called Planetary Boundaries [69, 70], within which there is a ‘Safe operating space for humanity’, whilst their transgression implies substantial global risks. Although an indicator for net chemical risks at the global scale has neither been formulated nor quantified as yet, it has triggered the development of two relevant concepts. These concepts can be put into practice by the development of the ‘model train’ to predict and quantify expected net mixture impacts for water bodies in Europe. One of these approaches is to aggregate all complex results into a single large-scale indicator, the chemical footprint. This approach is based on a fundamental principle of toxicology, dating back to Paracelsus, in short ‘all things are toxic; it is only the dose that makes a thing non-toxic’. The footprint approach is built upon an assessment of the net emissions of chemicals in a region, their effects on the ecosystem and if this effect can be neutralised to a no- or acceptable risk level given the available water dilution volume of the region [71–73]. The unit of the chemical footprint is “*m* ^*3*^ *water needed to dilute the emissions to a no*- *or acceptable risk level*”, and can be used together with the actually available water volumes in a region as an easily communicated ratio and large scale indicator: values lower than one indicate sufficient safety for a studied area, and above one indicates an increased risk for chemical impacts. The choice of indicator does not suggest that the primary solution to the potential problems is dilution but that the result of a considered abatement strategy (involving a set of technical and non-technical measures) can be expressed in terms of a lower footprint or a lowered ratio value (see Fig. 4). Vice versa, evaluation of scenarios for future chemical emission patterns could yield a larger footprint.

While the footprint can be derived using the full modelling train results for an area, the footprint approach specifically requires setting a boundary condition, to define the situation which is considered sufficiently safe. Currently, policies aimed at preventing effects of chemicals make use of such boundary conditions for (lack of) biodiversity impact, which are in turn defined via operational definitions such as the PNEC (Predicted No Effect Concentration for chemicals, e.g. in REACH) and water quality criteria for substances under the WFD. An important aspect in the future development of the chemical footprints is to explore the natural boundary of chemical pollution stress, while recognising the presence of variability of vulnerability differences across ecosystems and sites [74, 75]. Based on food web models derived from biomonitoring observations, it has been shown that the vulnerability of ecosystems to stress depends on ecosystem composition as well as the mode of action of a stressor, *i.e.* whether a stressor affects the system in a species way (e.g. the primary producers, or the top-carnivores) or randomly [76]. This observation has recently been translated into an approach to define the so-called ecosystem vulnerability distribution, as a method to account for ecosystem-specific boundary definition [72, 73]. The selected safe boundary will need be positioned at the safe side of the natural impact thresholds, as has earlier been done in the context of per-chemical risk limits like the PNEC.

The conceptual framework presented here was developed mainly from an ambition to structure and organise the complex assemblage of scientific-, political- and technical aspects of current and future management of chemical contamination of the aquatic environment. Since the focus is not only the current situation but also future chemicals management, the conceptual framework also describes activities where existing scenarios are used to predict which chemicals or groups of chemicals will be of importance in the years to come.

The scientific challenges to fill the conceptual framework with tools (analytical, bioanalytical), models and databases are considerable and the further progress within the on-going project SOLUTIONS and, e.g. the project *Ecological Key Factors* (EKF) focused on systems-oriented water quality assessments, based on principles, tools and models [77].

The conceptual framework presented here represents a first attempt to substantiate a set of key elements of- and to apply a new paradigm of “solutions-focused” management of chemicals. The future usefulness of the conceptual framework will to a large extent depend on the success of the science performed and our ability to fill the conceptual framework with practical knowledge and tools. Accessibility to information and data from monitoring and research as well as on uses and emissions in industry and products (today often not publically available) are also crucial for the further development of strategies for sustainable use of chemicals.
